# Parity and Overweight/Obesity in Peruvian Women

**DOI:** 10.5888/pcd14.160282

**Published:** 2017-10-19

**Authors:** Carlos A. Huayanay-Espinoza, Renato Quispe, Julio A. Poterico, Rodrigo M. Carrillo-Larco, Juan Carlos Bazo-Alvarez, J. Jaime Miranda

**Affiliations:** 1 CRONICAS Centro de Excelencia en Enfermedades Crónicas, Universidad Peruana Cayetano Heredia, Lima, Peru; 2 Department of Medicine, School of Medicine, Universidad Peruana Cayetano Heredia, Lima, Peru

## Abstract

**Introduction:**

The rise in noncommunicable diseases and their risk factors in developing countries may have changed or intensified the effect of parity on obesity. We aimed to assess this association in Peruvian women using data from a nationally representative survey.

**Methods:**

We used data from Peru’s Demographic and Health Survey, 2012. Parity was defined as the number of children ever born to a woman. We defined overweight as having a body mass index (BMI, kg/m^2^) of 25.0 to 29.9 and obesity as a BMI ≥30.0. Generalized linear models were used to evaluate the association between parity and BMI and BMI categories, by area of residence and age, adjusting for confounders.

**Results:**

Data from 16,082 women were analyzed. Mean parity was 2.25 (95% confidence interval [CI], 2.17–2.33) among rural women and 1.40 (95% CI, 1.36–1.43) among urban women. Mean BMI was 26.0 (standard deviation, 4.6). We found evidence of an association between parity and BMI, particularly in younger women; BMI was up to 4 units higher in rural areas and 2 units higher in urban areas. An association between parity and BMI categories was observed in rural areas as a gradient, being highest in younger women.

**Conclusion:**

We found a positive association between parity and overweight/obesity. This relationship was stronger in rural areas and among younger mothers.

## Introduction

Rates of overweight and obesity have risen dramatically during the last 3 decades, representing a global pandemic. In 2015, the burden of disease related to overweight and obesity accounted for 19.4 million deaths, 59 millions of years lived with disability (YLDs), and 411.6 millions of disability-adjusted life years (DALYs) (1). Rates of obesity have been leveling off in some highly industrialized societies, but rates of obesity remain high in the developing world (2–4).

Several well-known risk factors for obesity, including poor diet and low levels of physical activity, have been reported. Parity also appears to contribute to the prevalence of obesity (5,6). Some studies, using international data from up to year 2000, found a positive relationship between parity and obesity in all women in developed countries but only in the richest women in low-income and middle-income nations (7). Findings from studies from Brazil (8) and Chile (6) using data from 1996 and from 1997 and 1998, respectively, indicated a modest association between parity and obesity. Many developing countries are facing an increase in noncommunicable diseases (NCDs) and their risk factors, so the relationship between the risk factors for several NCDs could have changed or intensified — including the effect of parity on obesity (9).

Obesity rates in Peru have been escalating and vary according to area of residence and socioeconomic position (10). Peru had the highest fertility rate in the Latin American region in 2012 (11), and in the last decade, rates of women receiving adequate prenatal care services (ie, at least 4 visits to prenatal care) has been nearly 95% (11), indicating a major achievement of Peru’s health system. In this study, we aimed to 1) assess the relationship between parity and obesity in Peruvian women at the national level, 2) characterize this association by area of residence (urban and rural) and age, and 3) explore the distribution of nutritional status by parity in the country’s 24 administrative regions.

## Methods

### Study design and data set

We used data from Peru’s Demographic and Health Survey (DHS), 2012, a cross-sectional, nationally representative, multistaged, and probabilistic study that included rural and urban areas. Peru has 24 administrative regions and one constitutional department, Callao, which was evaluated together with Lima. The Human Development Index (HDI) for Peru’s 24 regions ranges from 0 to 1 (Appendix A). Values closer to 0 mean the country has a greater distance from the maximum achievable score; closer to 1 means the distance is shorter. HDI includes dimensions that address the well-being and development of the population and not just gross income. The dimensions included in the HDI are life expectancy (ie, health), education (eg, years of education), and standard of living (ie, income). In 2012, the regions with the highest HDI were Lima (0.63), Moquegua (0.62), Callao (0.59), and Arequipa (0.58).

DHS collects demographic and health information from women aged 15 to 49 years and children aged 5 years or younger who lived in the same household. In rural areas, villages of 500 to 2,000 people were the primary sampling unit, and households in each of these clusters were the secondary sampling unit. In urban areas, a primary sampling unit consisted of blocks or groups of blocks with more than 2,000 people and with 100 houses on average; secondary sampling units were the same as in rural settings. A total of 1,132 clusters and 27,709 households were part of this survey; the response rate was 99.1%. Additional information about the methods of Peru’s DHS is available (http://proyectos.inei.gob.pe/endes/).

Our original data set contained 26,172 observations. We excluded participants with missing information on body mass index (BMI) and the first child (n = 664). We also excluded women who were pregnant or breastfeeding at the time of the survey (n = 1,047), women who were widowed or divorced (n = 180), women with births in the last year (n = 1,775), and women without information for wealth index (n = 3,508) and education level (n = 2,916). In total, 10,090 records were excluded, giving a final number of 16,082 participants for the analysis.

### Variables

Parity was defined as the self-reported number of children ever born to a woman (7,12). We classified this variable into 3 categories: nulliparous (reference group), women with one child, and women with 2 or more children. We excluded data from women with children who were born during the last year because of possible pregnancy-related weight gain.

BMI (weight in kg divided by height in m^2^) was the main outcome. Weight was measured to the nearest 0.1 kg with participants wearing light clothing and no shoes, and height was measured to the nearest 0.1 cm. We used international standards to define normal weight (18.5–24.9), overweight (25.0–29.9), and obesity (≥30.0). We defined overweight/obesity as a BMI of 25.0 or higher and underweight as a BMI of less than 18.5.

Wealth index was divided into quintiles for each of the areas of residence, as used in a previous examination of a similar Peruvian survey (10). Years of education were constructed in quartiles. Education in years and wealth index quintile were adjusted by using weighting. Mothers’ age was separated into 3 categories (15–24 y, 25–34 y, and 35–49 y). Other variables collected by DHS, including those that were used as confounders, were urban/rural area of residence at the time of the survey, mothers’ age at first child, duration of breastfeeding in the last child younger than 5, type of delivery of last child younger than 5, current use of contraception, marital status, and frequency of watching television. We also included access to prenatal care to verify whether it changed according to parity and by urban/rural location; access to prenatal care was defined as the number of prenatal care visits (0, 1–3, and ≥4 visits) reported by the women. 

### Statistical analyses

Linear regression was performed by maximum likelihood estimation to assess the crude and adjusted (for confounding variables) association between BMI and parity. These models were run separately for subgroups of area of residence and age, after confirming an interaction effect using the Wald test. Prevalence ratios (PRs) were estimated separately for overweight, obesity and overweight/obesity (as binary outcomes) using log-Poisson models. PRs are preferred to odds ratios when outcome prevalence is high (13). We tested for effect modification for variables with biological plausibility as women’s age and residence using the Wald test (*P* < .05) for determining a statistical effect. We used maps to present the prevalence of overweight/obesity, overweight, and obesity, by parity status. For all analyses, we estimated 95% confidence intervals (CIs). We used Stata version 12.0 (StataCorp, LP) for all data analyses. Because of the survey’s design, all analyses were conducted using the *svy* command.

The National Institute of Statistics and Informatics (INEI) of Peru was responsible for obtaining informed consent from all participants for the survey information and anthropometric measures. We used anonymous data that are publicly available, so no institutional review board approval was required.

## Results

A total of 16,082 women were included in the analysis. Mean age of participants was 30.9 years (standard deviation [SD], 10.0 y), and mean BMI was 26.2 (SD, 4.6). The overall prevalence of overweight was 36.6% (95% CI, 35.6%–37.6%), and overall prevalence of obesity was 19.1% (95% CI, 18.2%–20.1%). For parity, 39.6% (95% CI, 38.5%–40.7%) were nulliparous, 17.1% (95% CI, 16.2%–18.0%) had one child, and 43.3% (95% CI, 42.3%–44.4%) had 2 or more children. Mean parity in rural women was 2.25 (95% CI, 2.17–2.33) and was 1.40 (95% CI, 1.36–1.43) in urban women. Table 1 shows demographic characteristics according to weight status in Peruvian women. We observed that the rate of access to prenatal care for younger women in rural areas was lower than for women in the older age groups in rural areas and for women of all age groups in urban areas. In urban areas, the rate of access to prenatal care was close to or greater than 95% (Appendix B).

**Table 1:** Sociodemographic Characteristics of Peruvian Women, by Weight Status, Peru’s Demographic and Health Survey, 2012^a^

Characteristic	Obs (16,082)	Underweight (n = 299)	Normal Weight (n = 6,799)	Overweight (n = 5,858)	Obese (n = 3,126)
		% (95% Confidence Interval)			
**Total children ever born**					
None	6,061	4.3 (3.6 to 5.0)	64.1 (62.5 to 65.8)	23.6 (22.2 to 25.1)	8.0 (7.0 to 9.0)
1	2,761	1.1 (0.5 to 1.7)	39.5 (37.1 to 41.9)	41.7 (39.2 to 44.3)	17.7 (15.6 to 19.7)
≥2	7,260	0.3 (0.2 to 0.5)	23.3 (21.9 to 24.7)	46.5 (45.0 to 47.9)	29.9 (28.2 to 31.5)
**Years of education, quartile**					
1st (Bottom)	4,537	1.7 (1.0 to 2.3)	32.3 (30.4 to 34.1)	40.5 (38.7 to 42.3)	25.6 (23.6 to 27.5)
2nd	6,537	2.7 (2.2 to 3.2)	46.7 (45.0 to 48.3)	33.6 (32.1 to 35.2)	17.0 (15.6 to 18.4)
3rd	1,458	2.0 (1.2 to 2.9)	50.8 (47.3 to 54.3)	32.9 (29.5 to 36.3)	14.3 (11.7 to 16.8)
4th (Top)	3,550	1.2 (0.8 to 1.7)	41.9 (39.7 to 44.1)	39.1 (37.0 to 41.2)	17.7 (15.8 to 19.7)
**Wealth index, quintile**					
1st (Bottom)	3,569	1.4 (1.0 to 1.9)	45.2 (43.0 to 47.4)	36.3 (34.4 to 38.3)	17.1 (15.4 to 18.7)
2nd	3,507	1.8 (1.2 to 2.3)	42.5 (40.2 to 44.7)	36.5 (34.5 to 38.6)	19.2 (17.3 to 21.2)
3rd	3,485	2.0 (1.4 to 2.7)	39.3 (37.1 to 41.5)	36.7 (34.5 to 38.9)	22.0 (20.0 to 23.9)
4th	3,072	2.3 (1.4 to 3.1)	41.2 (39.0 to 43.5)	37.6 (35.3 to 39.8)	18.9 (17.0 to 20.8)
5th (Top)	2,449	2.6 (1.8 to 3.4)	43.2 (40.7 to 45.8)	35.8 (33.4 to 38.3)	18.3 (16.1 to 20.6)
**Mother’s age, y**					
15–24	5,518	4.6 (3.8 to 5.4)	66.4 (64.8 to 68.1)	22.7 (21.3 to 24.2)	6.2 (5.2 to 7.2)
25–34	4,157	1.0 (0.5 to 1.5)	39.9 (37.9 to 42.0)	40.8 (38.8 to 42.7)	18.3 (16.6 to 19.9)
35–49	6,407	0.5 (0.3 to 0.7)	23.3 (21.8 to 24.7)	45.7 (44.0 to 47.3)	30.5 (28.7 to 32.3)
**Marital status**					
Never married	6,013	4.3 (3.6 to 5.0)	63.9 (62.2 to 65.6)	23.8 (22.3 to 25.2)	8.0 (7.0 to 9.1)
Not living together with a partner	1,675	1.6 (0.6 to 2.7)	32.6 (29.7 to 35.6)	37.7 (34.8 to 40.6)	28.0 (25.0 to 31.0)
Living together with a partner	4,775	0.4 (0.1 to 0.6)	29.4 (27.6 to 31.2)	46.1 (44.3 to 48.0)	24.1 (22.3 to 25.9)
Married and living together	3,619	0.3 (0.1 to 0.6)	24.8 (22.8 to 26.7)	46.8 (44.7 to 48.9)	28.1 (26.0 to 30.3)
**Type of area of residence**					
Urban	12,567	2.1 (1.8 to 2.5)	41.8 (40.6 to 43.0)	36.2 (35.1 to 37.3)	19.9 (18.8 to 21.0)
Rural	3,515	1.5 (1.0 to 2.0)	44.5 (42.4 to 46.7)	38.8 (37.0 to 40.6)	15.1 (13.5 to 16.7)
**Television viewing**					
None at all	264	0.3 (−0.3 to 0.8)	45.9 (38.2 to 53.6)	38.4 (30.7 to 46.0)	15.4 (9.8 to 21.1)
Less than or at least once a week	4,048	2.0 (1.4 to 2.5)	47.2 (44.9 to 49.4)	35.3 (33.3 to 37.3)	15.5 (13.9 to 17.2)
Almost every day	11,770	2.1 (1.7 to 2.4)	40.6 (39.4 to 41.7)	37.0 (35.8 to 38.2)	20.4 (19.2 to 21.5)
**Current use of contraception**					
None	8,519	3.2 (2.7 to 3.7)	51.2 (49.8 to 52.7)	30.0 (28.7 to 31.3)	15.6 (14.4 to 16.7)
Natural	1,759	0.6 (0.1 to 1.0)	32.6 (29.7 to 35.6)	44.6 (41.7 to 47.6)	22.1 (19.7 to 24.6)
Modern^b^	5,804	0.7 (0.3 to 1.0)	31.2 (29.5 to 33.0)	44.5 (42.8 to 46.1)	23.7 (22.0 to 25.3)
**Mother’s age when she had first child, y**					
No child	6,061	4.3 (3.6 to 5.0)	64.1 (62.5 to 65.8)	23.6 (22.2 to 25.1)	8.0 (7.0 to 9.0)
≤15	555	0.1 (0 to 0.2)	22.0 (17.4 to 26.5)	38.3 (32.8 to 43.8)	39.6 (33.5 to 45.7)
16–25	7,921	0.6 (0.3 to 0.9)	28.0 (26.6 to 29.4)	45.4 (44.1 to 46.8)	26.0 (24.6 to 27.4)
≥26	1,545	0.5 (0 to 0.9)	29.1 (26.0 to 32.3)	45.7 (42.3 to 49.0)	24.7 (21.7 to 27.8)
**Duration of breastfeeding, months, last child younger than 5 years**					
No breastfeeding	12,825	2.4 (2.0 to 2.7)	43.8 (42.6 to 45.0)	35.4 (34.3 to 36.5)	18.4 (17.5 to 19.4)
<6	201	0.6 (−0.5 to 1.7)	29.2 (20.4 to 38.0)	39.6 (31.3 to 47.8)	30.6 (21.8 to 39.5)
≥6	3,056	0.6 (0.2 to 0.9)	36.1 (33.8 to 38.3)	42.0 (39.8 to 44.3)	21.4 (19.3 to 23.4)
**Type of delivering of last child younger than 5 years^c^**					
No delivering	12,825	2.4 (2.0 to 2.7)	43.8 (42.6 to 45.0)	35.4 (34.3 to 36.5)	18.4 (17.5 to 19.4)
No caesarean section or natural	2,381	0.6 (0.3 to 1.0)	39.2 (36.7 to 41.6)	40.6 (38.1 to 43.1)	19.6 (17.2 to 21.9)
Caesarean section	876	0.4 (−0.2 to 1.0)	26.8 (22.8 to 30.9)	44.7 (40.7 to 48.8)	28.1 (24.0 to 32.1)

a Sampling, proportion, and confidence intervals by variable were calculated by using the expansion factor of the original sampling design of the survey.

b Contraception method modern included pill, intrauterine device, injection, condom, female or male sterilization.

c “No delivering” means that women do not have a child younger than 5 years to report a natural deliver or a C-section.

The relationship between BMI and age and area of residence was significant (*P* < .001). We found a positive association between parity and BMI in urban and rural areas across all age groups, except among women aged 35 to 49 years, in whom the only association found was for women in urban areas with 2 or more children (Table 2). This association was much stronger among women aged 15 to 24 years with 2 or more children than among nulliparous women. BMI was 4 units higher in young rural women and 2 units higher in young urban women. Most estimates were much higher in crude analyses after adjustment (Table 2).

**Table 2:** Relationship Between Parity and Body Mass Index in Peruvian Women (N = 16,082), by Age Group and Area of Residence, Peru’s Demographic and Health Survey, 2012^a^

Characteristic	Body Mass Index	
	Crude	Adjusted^a^
	β (95% Confidence Interval)	
**Urban Residence**		
**Aged 15–24 y**		
No child	1 [Reference]	
1 Child	1.96 (1.38 to 2.53)	1.46 (0.59 to 2.32)
≥2 Children	2.56 (1.73 to 3.38)	2.01 (0.67 to 3.35)
**Aged 25–34 y**		
No child	1 [Reference]	
1 Child	1.31 (0.81 to 1.80)	1.10 (0.55 to 1.64)
≥2 Children	2.40 (1.79 to 3.00)	1.96 (1.25 to 2.68)
**Aged 35–49 y**		
No child	1 [Reference]	
1 Child	0.78 (−0.04 to 1.61)	0.72 (−0.09 to 1.52)
≥2 Children	2.00 (1.24 to 2.75)	1.76 (0.97 to 2.54)
**Rural Residence**		
**Aged 15–24 y**		
No child	1 [Reference]	
1 Child	2.35 (1.75 to 2.94)	2.53 (1.38 to 3.68)
≥2 Children	3.60 (2.51 to 4.70)	4.01 (2.47 to 5.56)
**Aged 25–34 y**		
No child	1 [Reference]	
1 Child	1.48 (0.53 to 2.43)	1.52 (0.41 to 2.63)
≥2 Children	2.06 (1.02 to 3.10)	2.39 (0.98 to 3.81)
**Aged 35–49 y**		
No child	1 [Reference]	
1 Child	1.05 (−0.68 to 2.78)	0.39 (−1.34 to 2.12)
≥2 Children	1.65 (0.13 to 3.16)	0.98 (−0.59 to 2.54)

a Adjusted by education in years (in quartiles), wealth index (in quintiles), mother’s age in years, marital status, type of area of residence, television viewing, current use of contraception, mother's age when she had first child, duration of breastfeeding in last child younger than 5, type of delivery of last child younger than 5.

Interaction between binary outcomes of weight status (overweight, obesity, and overweight/obesity) and age and area of residence was significant (*P* < .001). In urban areas, an association between parity and BMI categories was observed only in women with 2 or more children in the oldest age group (Table 3). In rural areas, however, this association was observed in women with 2 or more children in 2 age groups (women aged 15–24 y and 25–34 y), being highest in younger women than older women (Table 3). In the youngest age group (aged 15–24 y), rural women with children had 4 to almost 7 times higher prevalence of obesity than nulliparous women.

**Table 3:** Prevalence Ratios for Parity and Overweight, Obesity, and Overweight/Obesity, by Age Group and Area of Residence of Mother, Peru’s Demographic and Health Survey, 2012^a^

Characteristic	Overweight		Obesity		Overweight/Obesity	
	Crude	Adjusted^a^	Crude	Adjusted^a^	Crude	Adjusted^a^
**Urban Residence**						
**Aged 15–24 y**						
No children	1 [Reference]					
1 Child	1.74 (1.45–2.08)	1.14 (0.77–1.68)	2.38 (1.56– 3.65)	0.77 (0.33–1.80)	1.70 (1.45–2.00)	1.08 (0.77–1.50)
≥2 Children	2.44 (1.92–3.08)	1.45 (0.90–2.34)	2.19 (1.25–3.86)	0.57 (0.17–1.95)	2.15 (1.75–2.64)	1.27 (0.83–1.95)
**Aged 25–34 y**						
No children	1 [Reference]					
1 Child	1.41 (1.22–1.63)	1.23 (0.96–1.58)	1.39 (1.08–1.81)	0.99 (0.60–1.63)	1.31 (1.17–1.46)	1.14 (0.94–1.39)
≥2 Children	1.56 (1.35–1.80)	1.31 (0.97–1.76)	2.16 (1.68–2.78)	1.32 (0.73–2.38)	1.49 (1.33–1.67)	1.23 (0.97–1.56)
**Aged 35–49 y**						
No children	1 [Reference]					
1 Child	1.27 (1.03–1.57)	1.21 (0.96–1.53)	1.35 (1.03–1.76)	1.18 (0.87–1.60)	1.20 (1.04–1.38)	1.14 (0.98–1.33)
≥2 Children	1.52 (1.26–1.83)	1.38 (1.09–1.74)	1.92 (1.51–2.45)	1.55 (1.13–2.13)	1.40 (1.23–1.58)	1.28 (1.09–1.49)
**Rural Residence**						
**Aged 15–24 y**						
No children	1 [Reference]					
1 Child	1.95 (1.54–2.47)	2.03 (1.18–3.51)	9.24 (5.12–16.66)	3.90 (1.13–13.43)	2.14 (1.75–2.60)	2.01 (1.28–3.16)
≥2 Children	2.50 (1.91 – 3.27)	2.96 (1.57–5.59)	12.95 (6.76–24.81)	6.57 (1.67 – 25.86)	2.66 (2.12–3.33)	2.68 (1.57–4.58)
**Aged 25–34 y**						
No children	1 [Reference]					
1 Child	1.13 (0.84–1.53)	1.44 (0.94–2.22)	2.09 (1.18–3.68)	2.37 (1.09–5.17)	1.22 (0.95–1.56)	1.53 (1.09–2.13)
≥2 Children	1.46 (1.13–1.88)	1.95 (1.09–3.50)	2.75 (1.62–4.68)	3.33 (1.34–8.29)	1.49 (1.20–1.84)	1.91 (1.23–2.95)
**Aged 35–49 y**						
No children	1 [Reference]					
1 Child	1.47 (0.97–2.22)	1.19 (0.67–2.12)	1.95 (0.90–4.22)	1.32 (0.51–3.43)	1.43 (1.02–2.01)	1.17 (0.73–1.87)
≥2 Children	1.61 (1.13–2.30)	1.14 (0.65–2.02)	2.45 (1.19–5.08)	1.36 (0.48–3.85)	1.57 (1.15–2.13)	1.17 (0.73–1.87)

a Adjusted by education in years (in quartiles), wealth index (in quintiles), mother’s age in years, marital status, type of area of residence, television viewing, current use of contraception, mother's age when she had first child, duration of breastfeeding in last child younger than 5, type of delivery of last child younger than 5.

In the analysis of regional prevalence, the highest rates of overweight/obesity in women with 2 or more children were found in Madre de Dios (87.3%), Tacna (86.0%), Moquegua (84.8%), Arequipa (84.1%), Ica (83.9%), La Libertad (83.2%), Ucayali (82.7%), and Lima y Callao (82.3%) (Figure).

**Figure:**
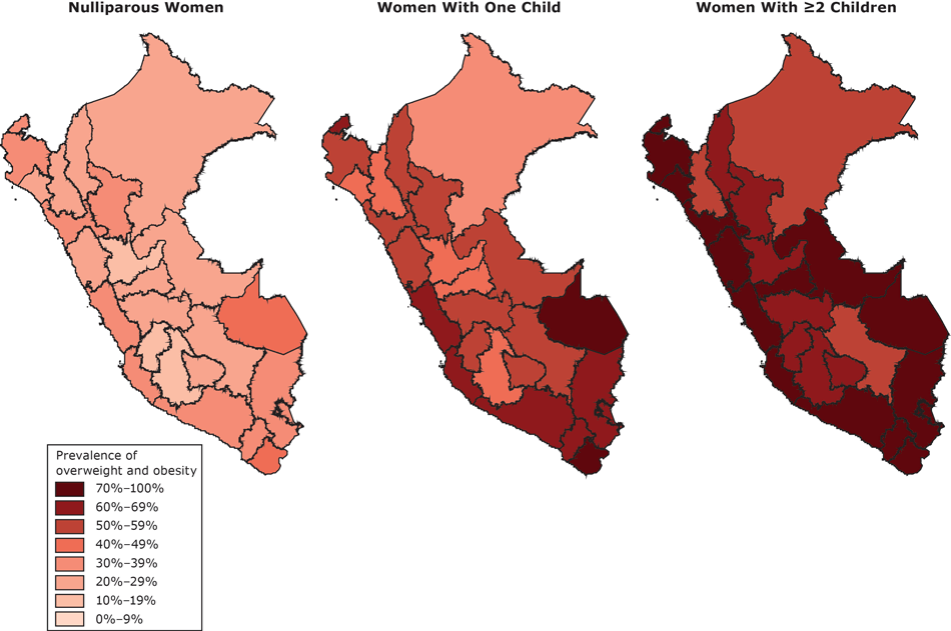
Regional prevalence of women with overweight/obesity, by number of children, Peru’s Demographic and Health Survey, 2012.

The prevalence of overweight and obesity in women with 2 or more children in all regions was greater than 38% and 15%, respectively. The highest obesity rates in women with 2 or more children were in Madre de Dios (45.1%), Moquegua (42.0%), Ica (37.6%), Tacna (37.6%), Arequipa (34.9%), Tumbes (33.0%), and Lima and Callao (32.4%). The prevalence of overweight in women with 2 or more children was highest in Pasco (50.5%), Lambayeque (49.8%), Ayacucho (49.7%), Ancash (49.6%), Ucayali (48.4%), and La Libertad (47.9%).

## Discussion

Peruvian women with more episodes of children born have a higher BMI as well as higher rates of overweight and obesity relative to their nulliparous counterparts, and this finding was more pronounced among young and rural women and especially those with 2 or more children. The youngest rural women with 2 or more children had a 4-times higher average BMI than did their nulliparous counterparts. The youngest urban women with 2 or more children had twice the average BMI of their counterparts without children. In terms of BMI categories, the association was more prominent in rural than in urban areas, with a clear gradient in the magnitude of the associations among childbearing women, higher in youngest women and lowest in oldest women. These findings signal potential avenues to start addressing obesity, especially in young childbearing women as well as women from rural areas, possibly adapting to and leveraging existing prenatal care initiatives.

Our findings align with results from previous studies on the association between parity and overweight/obesity in high-income countries (2) and in low-income and middle-income countries (3,4,6,8). This observation has been replicated by using waist circumference as the outcome (14). One study, using data from national surveys from 28 countries from 1996 through 2003, confirmed this association in countries with a high development index, but in least-developed countries this association was observed only among the richest women (7). Our study expands this knowledge to show a clearer pattern of worse overweight/obesity profiles in younger and rural childbearing women.

Developing countries, and especially their most vulnerable areas, are characterized by women with high levels of malnutrition. By contrast, in Peru, which is currently undergoing a rapid epidemiological transition, obesity has almost tripled in women aged 20 to 49, especially in rural areas (15). Our study findings show that parity, especially in young rural women, is an independent key factor in increasing rates of obesity. This population is also characterized by low levels of access to prenatal care among women in urban areas and among older women (Appendix B).

Conversely, rural health centers have historically focused on combating malnutrition and maternal death but not chronic NCDs. This fact was evidenced in 2015 by Michelle et al, who showed that the training programs of health professionals lack components linked to primary care (16). Moreover, these training programs are outdated because of current epidemiological changes (16). Similarly, a systematic review showed that pregnant women, especially those at risk for obesity, gestational diabetes, or malnutrition, do not receive adequate nutritional education, especially because of lack of time, resources, and training of health care providers (17).

Weight gain during pregnancy and at the puerperium could explain the parity-related obesity burden. Excess gestational weight gain could increase postpartum weight retention in the short term (18), with difficult and slow weight loss in the long term (5). Energy in excess of fetal needs may be stored given the anabolic condition of pregnancy, increasing the body’s fat percentage (19). Weight retention and its corresponding weight loss after birth skew and complicate measurement of the nutritional status of the mother. Other pregnancy and puerperal conditions may also play a role in the development of obesity in women of childbearing age. Some conditions — such as increasing physical inactivity and unhealthy dietary patterns (20) — may occur months after the end of pregnancy and others throughout the lifespan. Lifestyle changes lead this process, where sedentary lifestyles are increasing and unhealthy diets have dramatically Westernized in rapidly transitioning societies like Peru (10,15).

Excess maternal weight during pregnancy may be associated with overweight and obesity, as well as risk of postpartum diabetes (22), in the mother’s life (21). Conversely, early pregnancy may lead to a higher probability of obesity (23), predominantly abdominal, a significant risk factor for chronic diseases progression (24). Women’s BMI, weight gain during pregnancy, and parity could also affect the health of offspring, predisposing children to overweight or obesity in young adulthood (25). All of these consequences can be avoided with preventive interventions, especially in young women, focused on monitoring and promoting healthy weight during and after pregnancy (26).

Obesity in Peruvian women has been linked to high levels of wealth, low levels of education, and urban contexts (10). Other factors are consumption of high-fat, carbohydrate-dense foods and physical inactivity, which could affect obesity rates and differences in rates by country, in both rich and poor subgroups (27). We found a positive and strong association between parity and obesity, mainly in young and rural women.

In Peru, the obesity and overweight rates found at the regional level were similar to those reported previously (28). The prevalence was higher than 50% in women with 2 or more children in all regions of the country. These findings should guide future prevention strategies, with priority given to the regions with the highest prevalence of obesity, such as Madre de Dios, Moquegua, and Arequipa, and mainly in populations of young women in rural areas.

Our results show the importance of focusing efforts on family planning and on prenatal and postnatal control, with a focus on nutrition not only of the children but also of the mother. Women’s rights and freedom of choice must also be addressed to avoid ethical issues that have occurred in the past decades.

This study has strengths and limitations. We used a nationally representative data set, and even after exclusion of observations caused by incomplete information or eligibility criteria, our study benefited from a large sample size that allowed for disaggregated analyses by rural and urban area and age. We also had data on total number of births, not only number of children alive, in our data set compared with other studies (12). Furthermore, this study presents novel findings about the effect modification of area of residence and age. However, we cannot infer causality because of the cross-sectional design of our study.

Another limitation was the inability to measure BMI before and after pregnancy and for subsequent pregnancies (29). Prepregnancy BMI is recognized as a key factor in the parity–overweight/obesity relationship (8). Weight gain after pregnancy could be higher in women with a high prepregnancy BMI, and the effect of lactation is another factor that could help women to control their weight (8). We lacked information about lifestyle, such as diet and physical activity, especially in rural areas. The consumption of *trans*-fatty acids and low levels of physical activity during the postpartum period increases the likelihood of overweight and obesity (20,30).

Despite these limitations, this study analyzed the relationship between parity and obesity using several confounding factors that explain and contextualize this relationship in a geographically diverse country that spans coastal, Andean mountains, and jungle areas.

Data from our nationwide survey confirm a positive parity–obesity association of varying magnitudes, provide a detailed within-country regional characterization of the overweight/obesity–parity relationship, and show that younger and rural childbearing women carry a higher risk of overweight and, in particular, obesity.

Interventions aimed at promoting better nutritional status before and during the gestational period should be encouraged, as should promoting maternal health in a community with rising rates of NCDs, by addressing obesity through existing and ongoing reproductive health programs. Strategies to follow and supervise women in the gestational and breastfeeding periods for weight control should be encouraged. The challenge is to strengthen primary health care in maternal and neonatal services, with an emphasis on the prevention of chronic NCDs, especially in young rural women.

## Appendices

### Appendix A. Region by Human Development Index, 2012. Source: PNUD Peru (based on National Institute of Statistics and Information, Ministry of Finance, Ministry of Education of Peru)

**Table:**

Region	Human Development Index
Huancavelica	0.2962
Ayacucho	0.3336
Apurímac	0.3444
Huánuco	0.3746
Cajamarca	0.3773
Amazonas	0.3846
Puno	0.3942
Loreto	0.3977
Pasco	0.4114
Ucayali	0.4324
Piura	0.4379
San Martin	0.4408
Ancash	0.4429
Cusco	0.4434
Junín	0.4539
Lambayeque	0.4617
La Libertad	0.4653
Tumbes	0.5184
Ica	0.5351
Tacna	0.5553
Madre de Dios	0.5582
Arequipa	0.5781
Callao	0.5863
Moquegua	0.6215
Lima	0.634

### Appendix B. Prenatal Control Number According to Age Group

**Table:**

Age Groupᵃ	Women With 1 Child, %			*P* Value	Women With ≥2 Children			*P* Value
	No Controls	1–3	≥4		No Controls	1–3	≥4	
**Urban and rural areas**								
15–24	1.07	6.28	92.65	<.001	1.28	8.56	90.15	.14
25–34	0.16	2.31	97.53		0.54	4.13	95.34	
35–49	0.23	1.41	98.36		0.57	5.59	93.84	
**Urban areas**								
15–24	0.83	5.22	93.95	<.001	0.79	8.51	90.7	.12
25–34	0.18	2.31	97.5		0.56	4.54	94.89	
35–49	0.26	0.3	99.45		0.57	5.44	93.98	
**Rural areas**								
15–24	1.88	9.72	88.4	.19	2.72	8.72	88.57	.03
25–34	0	2.3	97.69		0.42	2.22	97.36	
35–49	0	12.37	87.63		0.56	6.45	92.99	

ᵃ Proportion by variable was calculated by using the expansion factor of the original sampling design of the survey.
